# Intramesenteric abscess caused by non-typhoidal *Salmonella*

**DOI:** 10.1016/j.idcr.2022.e01523

**Published:** 2022-06-06

**Authors:** Connie Au, Shannon Skochko, Anthony Hung Chau

**Affiliations:** a University of California Irvine School of Medicine, Irvine, CA, USA; b University of California Irvine, Department of Surgery, Orange, CA, USA; c VA Long Beach Health Care System, Long Beach, CA, USA

**Keywords:** Salmonella, Abscess, Intraabdominal infection, Surgical drainage

## Abstract

Nontyphoidal *Salmonella* (NTS) is a common cause of gastroenteritis in humans and animals, but intra-abdominal abscesses or organ space surgical site infection (SSI) secondary to this organism has been rarely reported, making diagnosis and management difficult. Our case of intra-mesenteric abscess caused by NTS species is the only case reported in the literature. Immunocompromising conditions such as diabetes and human immunodeficiency virus (HIV) are important risk factors for invasive nontyphoidal *Salmonella*. Most patients are treated initially with intravenous antibiotics. Treatment often requires operative drainage by laparoscopy or laparotomy alone, although percutaneous drainage has been performed more frequently in recent years. Early clinical signs and radiographic features of intra-abdominal abscess may be diagnostically challenging. It is important for clinicians to have high index of suspicion based on history and symptomatology as prompt treatment is essential to prevent further morbidity and mortality.

## Introduction

Nontyphoidal *Salmonella* (NTS) causes approximately 153 million cases of gastroenteritis and 57,000 deaths globally each year [1]. In industrialized nations, NTS is transmitted predominantly by commercial food products contaminated by animal feces, often causing large food outbreaks [2]. Infection may also be spread via direct contact with an infected animal or between humans [1].

NTS primarily causes self-limited gastroenteritis symptoms of diarrhea, abdominal pain, fever, and vomiting [1], [3]. However, in approximately 8 % of cases, it can disseminate beyond the gastrointestinal (GI) tract via the vascular or lymphatic system, progressing to bacteriemia, pericarditis, meningitis, osteomyelitis, and abscess formation [1], [3]. Abscesses caused by NTS have been found in numerous organs and cavities including the spleen, liver, thyroid, ovaries, skin, and retroperitoneal space [3]. Immunocompromising conditions including diabetes, human immunodeficiency virus (HIV), sickle cell disease, and renal disease increase the risk for invasive nontyphoidal salmonella infections [3], [4], [5]. In this paper, we present a rare case of an intra-mesenteric abscess caused by *Salmonella Thompson* in a diabetic patient.

*Salmonella Thompson* is a salmonella serotype that has been implicated in numerous food-borne disease outbreaks throughout the world [6]. It has been found in a wide range of products, such as poultry, seafood, contaminated coriander, lettuce, and pet treats [6]. Data from the U.S. Food and Drug Administration shows *S. Thompson* to be the seventh most common serotype found in imported seafood [7]. Patients with *S. Thompson* infection rarely develop complications, typically reporting diarrhea, abdominal pain, fever, and vomiting [8].

## Case report

**Fig. 1:**
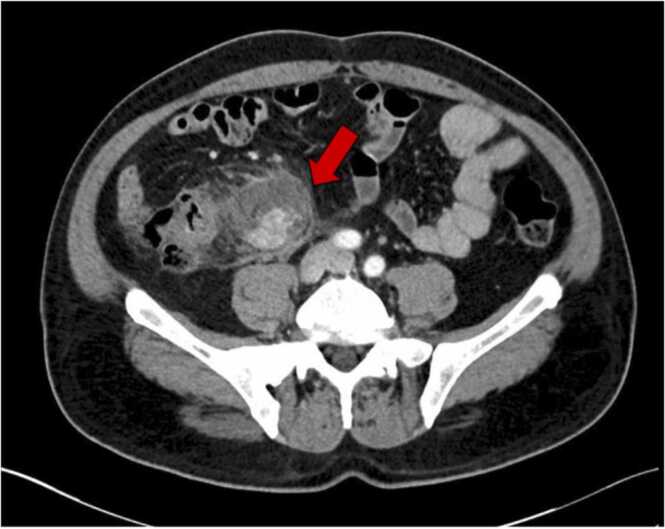
Initial CT abdomen/pelvis. Red arrow indicates an ill-defined 5–6 cm focal fluid collection with surrounding inflammation in the right mid abdomen center around what appears to be a ruptured aneurysm or pseudoaneurysm.

A 53-year-old male with a past medical history of diabetes mellitus presented as an interfacility transfer for suspected ruptured superior mesenteric artery (SMA). The patient reported a 2-week history of constipation and diffuse, constant abdominal pain, failing to resolve with laxative use. At the outside hospital a computed tomography (CT) abdomen/pelvis with intravenous (IV) contrast was performed and was remarkable for a five-to-six-centimeter contrast containing fluid collection with surrounding inflammation in the right mid abdomen concerning for ruptured SMA aneurysm (Fig. 1).

On arrival to our institution, he was afebrile, normotensive and heart rate 80 beats per minute, normal respiratory rate and oxygen saturation. On exam, his abdomen was soft with moderate diffuse tenderness to palpation and bilaterally palpable femoral pulses. Labs were notable for hemoglobin 12.3 (g/DL), white blood cell count 13.0 (10^9^/L) and lactic acid 0.8 (mEq/L).

The patient was taken to the operating room (OR) for emergent aortoiliac and SMA angiogram. SMA angiogram demonstrated normal branching of the proximal and distal SMA, and angiogram of the ileocolic and its branches did not reveal any active extravasation. No SMA aneurysm was noted. An immediate diagnostic laparoscopy did not reveal any peritoneal findings. The patient was subsequently admitted to the medical/surgical unit where he was initiated on IV ceftriaxone and metronidazole for presumed sepsis secondary to a right lower quadrant (RLQ) abscess. Blood cultures were obtained prior to antibiotic initiation and did not show evidence of bacteremia. A colonoscopy was performed which did not yield any colonic pathology.

**Fig. 2:**
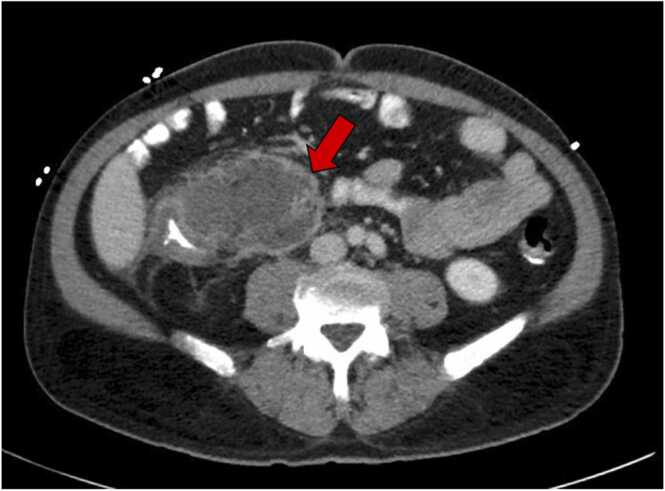
Repeat CT abdomen/pelvis on hospital day 9. Red arrow indicates a large 8.4 cm complex collection with surrounding mesenteric edema in the right lower quadrant is contiguous with the cecum, ascending colon, and terminal ileum.

On hospital day nine, repeat CT abdomen/pelvis was performed due to the patient’s inability to tolerate PO intake and demonstrated a progressive 8.4 cm complex collection with surrounding mesenteric edema in the RLQ contiguous with the cecum, ascending colon, and terminal ileum and suggestive of an abscess or arteriovenous malformation (Fig. 2). Repeat abdominal angiography performed by interventional radiology was unremarkable for intra- abscess extravasation.

Given the patient’s persistent abdominal pain and intolerance to diet, the patient was taken to the OR for exploratory laparotomy on hospital day ten. A large intra-mesenteric abscess abutting the cecum and right colon was drained and a drain was placed. The patient was continued on IV antibiotics and discharged on post-operative day eight. Operative cultures were positive for *Salmonella thompson*, and the patient was transitioned to oral levofloxacin for a four-week course per recommendations by our infectious disease consultant. The patient completed his antibiotic course at home. He was evaluated in the clinic four weeks postoperatively with complete resolution of his pain and has regained his bowel function.

## Discussion

We describe the first reported case of intra-mesenteric abscess caused by NTS. Although primarily confined to the GI tract, NTS may cause extra-intestinal infection via systemic spread or local invasion. NTS may spread systemically by invading the bloodstream causing bacteremia or propagating into the lymphatic system, contributing to remote abscess formation of the pathogen in various organs [3], [9], [10], [11]. Additionally, the bacterium may survive inside of macrophages for an extended period of time and create a chronic carrier state that can result in distal infection long after acute illness [3], [9], [11], [12]. The organism has also been shown to disseminate locally around areas with previous trauma or preexisting anatomical abnormalities [3], [13].

Immunocompromising conditions such as diabetes and HIV are important risk factors for invasive NTS infection. In one case-control study, patients who developed an infection after exposure to an *S. enteritidis*-contaminated meal were more likely to be medication-dependent diabetics [14]. Opportunistic infection due to diabetes is likely a result of decreased gastric acidity and autonomic neuropathy of the GI tract that reduces bowel movement and prolongs transit time [9], [14].

The exact etiology in our case of intra-mesenteric abscess is unknown. We speculate our patient likely had a primary GI infection given his two-week history of diffuse abdominal pain. The pathogen likely invaded and proliferated within the mesenteric lymph nodes, forming an abscess. Blood cultures acquired prior to antibiotic administration show no growth, providing lower suspicion for *Salmonella* bacteremia. There was also no history of open trauma or anatomic abnormalities seen in surgery that may have contributed to abscess formation.

Intraabdominal abscesses can be difficult to diagnose based on clinical presentation alone. In a study of 50 patients with intraabdominal *Salmonella* infection, the mean duration of symptoms before diagnosis was 13 days [3]. A majority of these patients presented with fever, abdominal tenderness, and palpable mass or organomegaly [3]. Imaging with CT or ultrasound (US) is often used to aid in diagnosis and localize area of infection. CT has been shown to have higher sensitivity, specificity, accuracy, and negative and positive predictive values than US in the detection of intra-abdominal abscess and should be the preferred initial imaging modality [15]. As highlighted by our case, early radiographic appearance of intra-abdominal abscess may be diagnostically challenging highlighting the importance for clinicians to have high index of suspicion based on history and symptomatology.

The Surgeon Infection Society and the Infectious Diseases Society of America have released evidence-based guidelines for the treatment of intraabdominal infection [16], [17]. Before initiation of treatment, it is important to perform a risk assessment to determine the possibility of mortality and treatment failure [16]. Determining risk of adverse outcomes can help guide decisions regarding source control and antibiotic management.

The primary management of intraabdominal infections involves source control procedures to remove infected fluid and tissue and/or antimicrobial treatment to target sites of infection and prevent spread or recurrence [3], [16], [17]. It is recommended to initiate empiric antimicrobial therapy within one hour upon diagnosis of intraabdominal infection [16]. Empiric antimicrobial regimen should target for typical gram-negative Enterobacteriaceae, gram-positive cocci, and anaerobes [16], [17]. Patients with abscess diameter more than 6.5 centimeters (cm) and temperature at admission more than 101.2 Fahrenheit are more likely to fail conservative therapy with antibiotics alone [18]. If the symptoms of infection do not resolve with antimicrobial treatment, source control procedures such as percutaneous abscess drainage or surgical laparotomy may be necessary [16], [17].

Selection of source control procedure should be predicated on careful assessment of the patient’s physiologic function and optimization of infection management [16]. Percutaneous radiology-guided drainage can be used to cure infection or improve patient status prior to operation [19]. The procedure is minimally invasive and has a success rate of over 80 % [16], [18], [19]. Compared to open surgical drainage, percutaneous drainage is associated with lower mortality even after controlling for severity of illness and should be the preferred initial treatment for source control in eligible patients [20].

NTS intraabdominal infections can be effectively treated with ampicillin, amoxicillin, trimethoprim-sulfamethoxazole, and chloramphenicol after appropriate source control [3]. However, recent food outbreaks of antibiotic resistant NTS have been reported in increasing frequency [21]. Cultures of drained abscess fluid should be obtained to optimize antimicrobial therapy for high risk patients [16]. De-escalation of empiric antibiotic therapy after determining pathogen sensitivity is recommended to limit overuse of broad-spectrum antibiotics and prevent antibiotic resistance [16], [17]. Recent studies have shown effective treatment with 4–7 days of antimicrobial therapy for intraabdominal infections and bacteremia [16], [17]. However, patients taking concurrent immunosuppressive medications or those with ongoing infection may require prolonged antibiotic treatment [16].

Prognosis for *Salmonella* intraabdominal infections is favorable, with one study reporting 90 % survival in a study of 50 patients [3]. Survival rate varies depending on the location of infection and abscess formation. Patients with NTS infected splenic or liver abscesses have higher mortality compared to cholecystitis caused by NTS infection [3]. In this case, our patient saw gradual improvement of abdominal pain and was safely discharged with oral antibiotics.

## Conclusion

NTS infection is commonly associated with gastroenteritis, but in 8 % of cases, it may present as extra-intestinal infection [1], [3]. NTS may be a source of abscesses in numerous organs and body cavities, including the spleen, liver, pancreas, ovaries, kidneys, and adrenal glands. To our knowledge, we present the only case of intra-mesenteric abscess caused by NTS species. Diagnosis of NTS intra-abdominal abscess can be difficult to diagnose clinically, often requiring CT imaging to localize area of infection. Treatment includes source control procedures such as surgical or percutaneous drainage and antibiotic therapy. Overall, prognosis of intraabdominal NTS infections is favorable when diagnosed promptly and treated appropriately.

## Declaration of Competing Interest

The authors report no conflict of interest or funding sources.
